# Distal aortic replacement followed by endovascular repair for the management of severe intra-pleural adhesions accidentally detected during open surgery for chronic type B aortic dissection: a report of two cases

**DOI:** 10.1186/s13019-022-02002-6

**Published:** 2022-10-08

**Authors:** Masahiro Daimon, Ryo Shimada, Yoshikazu Motohashi, Hiroaki Uchida, Hideki Ozawa, Takahiro Katsumata

**Affiliations:** Department of Thoracic and Cardiovascular Surgery, Osaka Medical and Pharmaceutical University, 2-7, Daigakumachi, Takatsuki, Osaka 569-8686 Japan

**Keywords:** Chronic type B aortic dissection, Thoracic endovascular aortic repair, Intra-pleural adhesion, Thoracotomy, Case report, Open repair

## Abstract

**Background:**

Open repair is the most promising curative treatment option for patients with chronic type B aortic dissection. However, based on our experience, following the accidental detection of intra-pleural adhesions during open surgery for chronic type B aortic dissection, complete replacement of the diseased aorta cannot be accomplished. To overcome this problem, we switched the procedure to create a distal landing zone for subsequent endovascular repair by replacing the distal aorta with a vascular graft.

**Case presentation:**

We report two cases in which open repair was attempted; however, the proximal descending thoracic aorta could not be exposed due to the presence of severe adhesion in the pleural cavity. In these patients, we accessed the lower descending thoracic aorta or thoracoabdominal aorta and created a distal landing zone for subsequent endovascular repair by replacing the aorta with a vascular graft. Thereafter, endovascular repair was performed with good outcomes.

**Conclusions:**

Replacement of the distal aorta, which is typically easy to access despite the presence of intra-pleural adhesions, with a vascular graft serves as a reliable distal landing zone for subsequent endovascular repair. This method may be a viable option for the management of severe adhesions accidentally detected in the pleural cavity during open repair for chronic type B aortic dissection.

## Background

Open aortic repair remains the most promising curative treatment option for patients with chronic type B aortic dissection (cTBAD) [1]. However, though rare, severe adhesions may be detected in the pleural cavity during open surgery for cTBAD. In such cases, thoracic endovascular aortic repair (TEVAR) may be the next best option.

Although it has been reported that TEVAR is useful in some cases with cTBAD, its indication in this setting remains controversial [2]. In particular, the lack of an appropriate distal landing zone is one of the reasons for the failure of TEVAR in patients with cTBAD.

Herein, we report our experience with two cases, in which open repair was attempted. However, the proximal descending thoracic aorta could not be exposed due to the presence of severe adhesions in the pleural cavity. In these patients, we accessed the lower descending thoracic aorta or thoracoabdominal aorta and created a distal landing zone for subsequent endovascular repair by replacing the aorta with a vascular graft. Thereafter, TEVAR was performed and yielded good outcomes.

## Case presentation

### Case 1

**Fig. 1:**
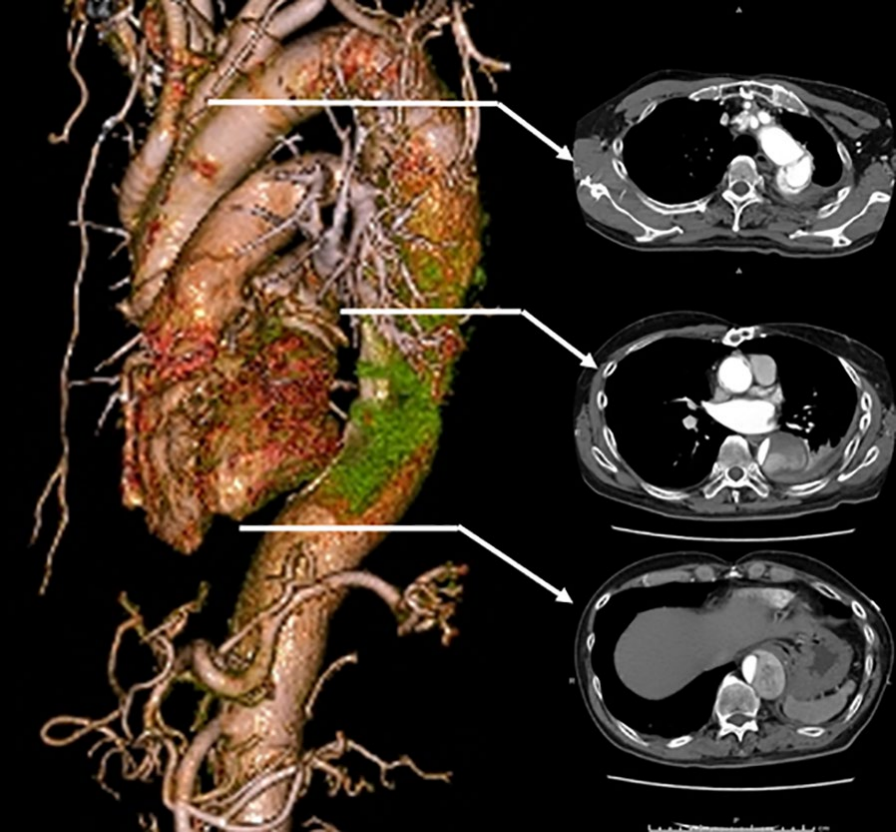
Enhanced computed tomography imaging. A preoperative CT scan showed aneurysmal dilatation of the mid descending thoracic aorta with a maximum diameter of 54 mm

A 61-year-old man with a history of acute type A aortic dissection 5 years earlier, in which the ascending, arch, and proximal descending aorta were replaced using a 22-mm four-branched vascular prosthesis (Gelweave, Vascutek Terumo Inc., Scotland, UK) via a median sternotomy with a left thoracotomy (the ‘trapdoor thoracotomy’) [3]. The elephant trunk method was not applied. At that time, bleeding from the distal aortic anastomosis was observed. Consequently, a wide felt strip was wrapped around the anastomosis to attain hemostasis. This patient also had left hemiparesis due to postoperative cerebral infarction. The patient was referred to our institute for the treatment of aneurysmal dilatation of the remaining descending thoracic aorta. Dissection was observed from the distal anastomosis of the vascular graft to the abdominal aorta. A preoperative thoracoabdominal computed tomography (CT) scan is showed aneurysmal dilatation of the mid descending thoracic aorta (Fig. 1). There was no obvious detection of Adamkiewicz's artery; however, the left side of the Th12 intercostal artery was considered critical.

A thoracotomy was performed at the left fourth intercostal space, revealing severe adhesions throughout the left lung above the hilar region caused by the previous thoracotomy. Moreover, the previous vascular graft and aneurysmal descending aorta could not be exposed. Thus, complete replacement of the diseased segment was impossible. Therefore, we decided to create a distal landing zone in preparation for a subsequent TEVAR, because the lower descending thoracic aorta was exposable with relatively mild adhesion to the lung.

**Fig. 2:**
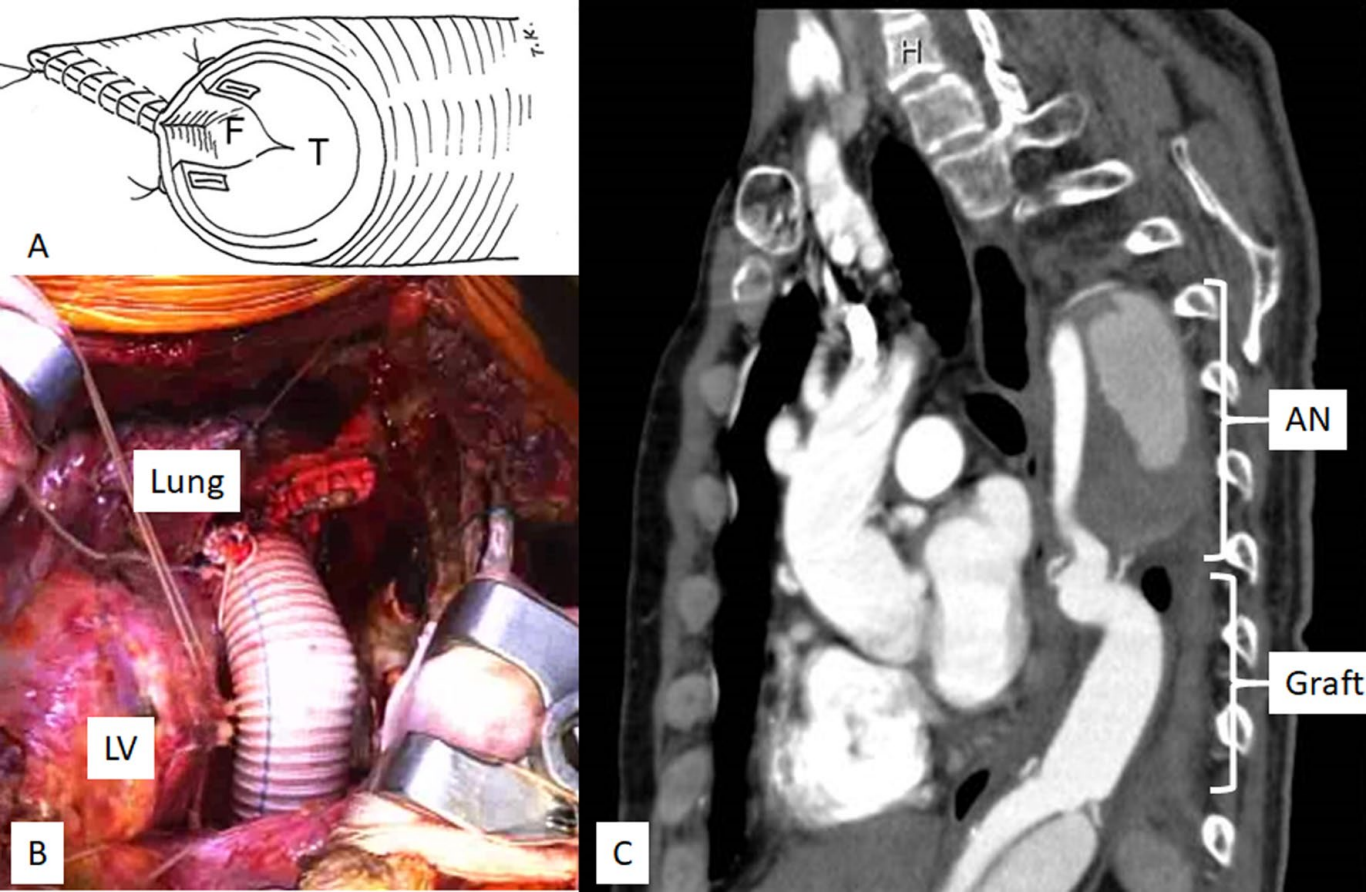
The schema shows the methodology used to reduce the size of the false lumen through double-barrel aortic anastomosis in the dissecting aneurysm (**A**). Intra-operative view (**B**) thorough the left fourth intercostal thoracotomy and postoperative enhanced computed tomography imaging (**C**). The descending thoracic aorta was replaced at a level of Th8 to Th10. AN, aneurysm; F, false lumen; LV, left ventricle; T, true lumen

The fifth intercostal space was opened through the same skin incision. The relatively mild adhesions in the lower lobe of the lung facilitated exposure of the distal descending thoracic aorta. Extracorporeal circulation was established by left femoral artery perfusion with right atrium drainage via the right femoral vein. Rectal temperature was cooled to 20 °C. Under deep hypothermic ventricular fibrillation (Vf), aortic cross-clamping was performed at the level of Th10. Next, the descending thoracic aorta was opened proximally under hypothermic lower body circulation. At this time, the patient was placed in the Trendelenburg position, and the central venous pressure was mildly increased (4–5 mmHg) to maintain retrograde cerebral circulation. The aorta was completely transected at the level of Th8 and anastomosed with a 22-mm branched vascular prosthesis, namely J-graft SHIELD NEO (Japan Lifeline Co., Tokyo, Japan). A double-barrel anastomosis was required because the true lumen was small (10 mm × 18 mm). The false lumen enlarged to 45 mm was obliterated laterally with two felt strips in a sandwich fashion to match the size of the prosthesis, and the intimal flap was incised longitudinally (Fig. 2A). After completion of the proximal anastomosis, upper body perfusion was resumed from a branch of the prosthesis. Under lower body circulatory arrest, the descending aorta was opened distally and completely transected at the level of Th10. Subsequently, open distal anastomosis was performed to the true lumen of the aorta (Fig. 2B). The vascular prosthesis was covered by the pleura. The cardiopulmonary bypass, hypothermic Vf, retrograde cerebral perfusion, and operation times were 209, 140, 47, and 551 min, respectively. A postoperative CT scan was performed after the operation (Fig. 2C).

Sixteen days later, TEVAR was performed through the right common femoral artery. A 34 × 200 mm Conformable TAG (W. L. Gore & Associates, Inc., Flagstaff, AZ, USA) was deployed in the aortic arch previously replaced with the 22-mm vascular prosthesis just distal to the branch of the left subclavian artery. A 34 × 150-mm Conformable TAG was subsequently deployed in the first stent graft to the distal 22-mm vascular prosthesis with a 3-cm overlap.

**Fig. 3:**
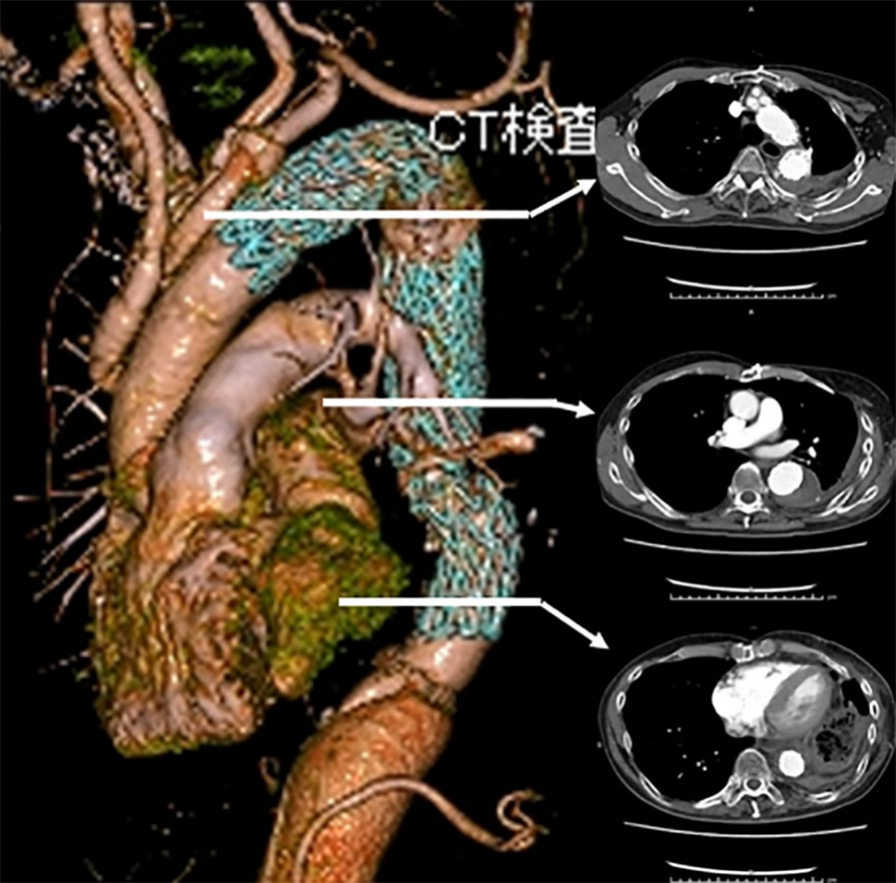
Enhanced computed tomography imaging. A postoperative CT scan did not show signs of further enlargement of the descending thoracic aorta with a completely thrombosed false lumen

The patient had an uneventful clinical course and was discharged from hospital 17 days after TEVAR. Seven years after the procedure, the patient has no signs of enlargement of the descending thoracic aorta (Fig. 3).

### Case 2

**Fig. 4:**
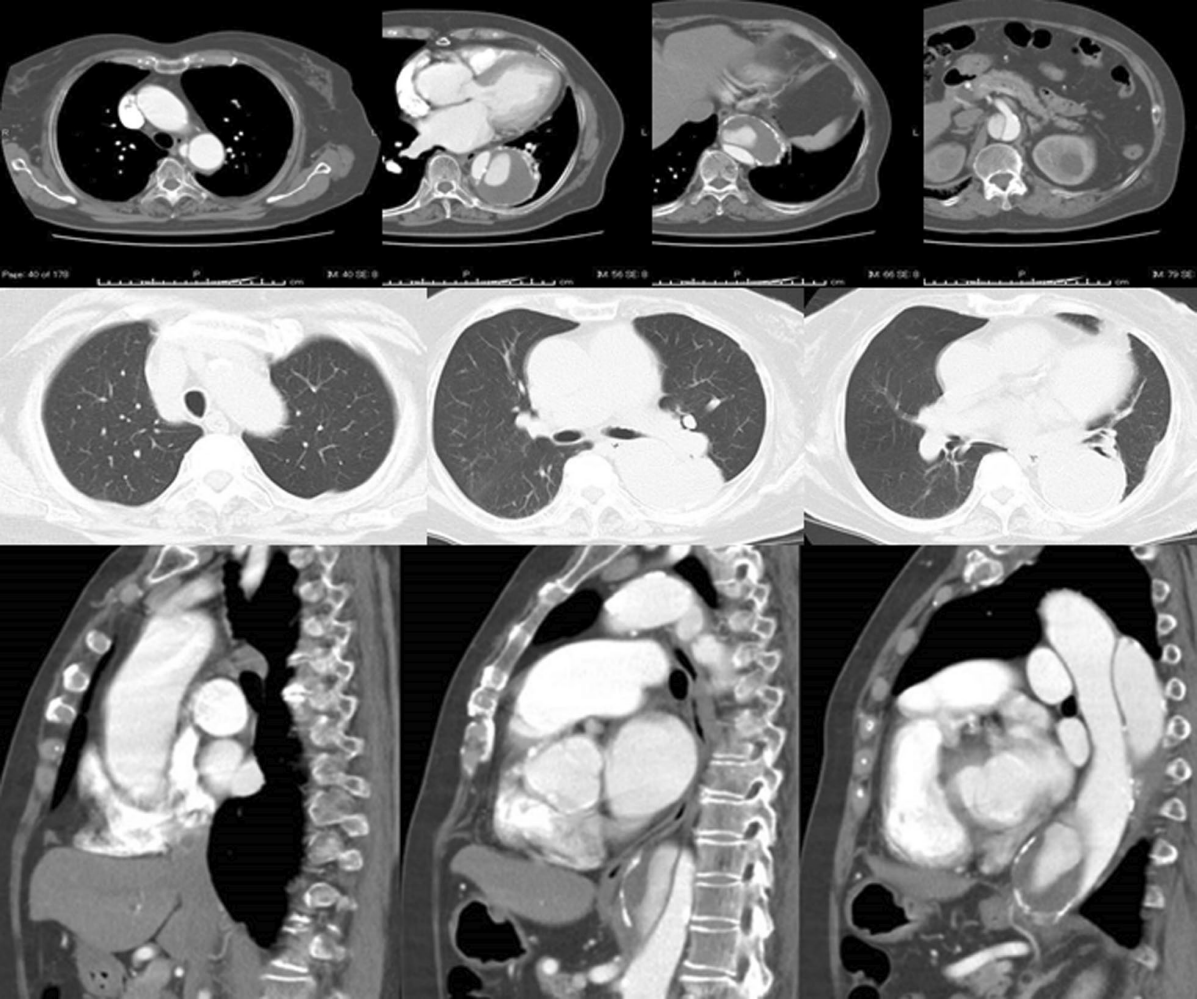
Upper: Enhanced computed tomography imaging. A preoperative CT scan showed chronic type B aortic dissection with a partially thrombosed false lumen. Calcification of the aortic wall on the distal thoracic aorta was observed. The maximum diameter of the aorta was 6.5 cm. Middle: The lung window setting showed no significant findings. Lower: Sagittal view of the enhanced CT imaging

An 80-year-old woman with a history of acute type B dissection 8 years earlier, treated with optimal medical therapy. During follow-up, she presented with enlarged descending thoracic aorta and was referred for surgery. The patient had a history of pneumonia of unknown details in her childhood and coronavirus-2019 infection 8 months earlier. A preoperative thoracoabdominal CT scan showed enlargement of the descending thoracic aorta with a partially thrombosed false lumen and calcification of the aortic wall on the distal descending aorta (Fig. 4). The presence of Adamkiewicz’s artery was not detected preoperatively.

A Crawford type I thoracoabdominal aortic replacement was planned. A lateral thoracotomy was performed at the sixth intercostal space, revealing the presence of severe adhesions in the pleural cavity. The adhesions around the upper lobes of the lung were particularly severe, and the risk of lung injury during dissection of the adhesion was extremely high. Thus, it was impossible to expose the distal aortic arch and proximal descending thoracic aorta. Therefore, as in Case 1, we decided to create a distal landing zone in preparation for subsequent TEVAR by performing a Crawford type IV replacement.

The costal arch was dissected, the diaphragm was separated, and the abdominal aorta was exposed through the retroperitoneal approach. The adhesiotomy around the lung was facilitated by the dissection of the costal arch.

**Fig. 5:**
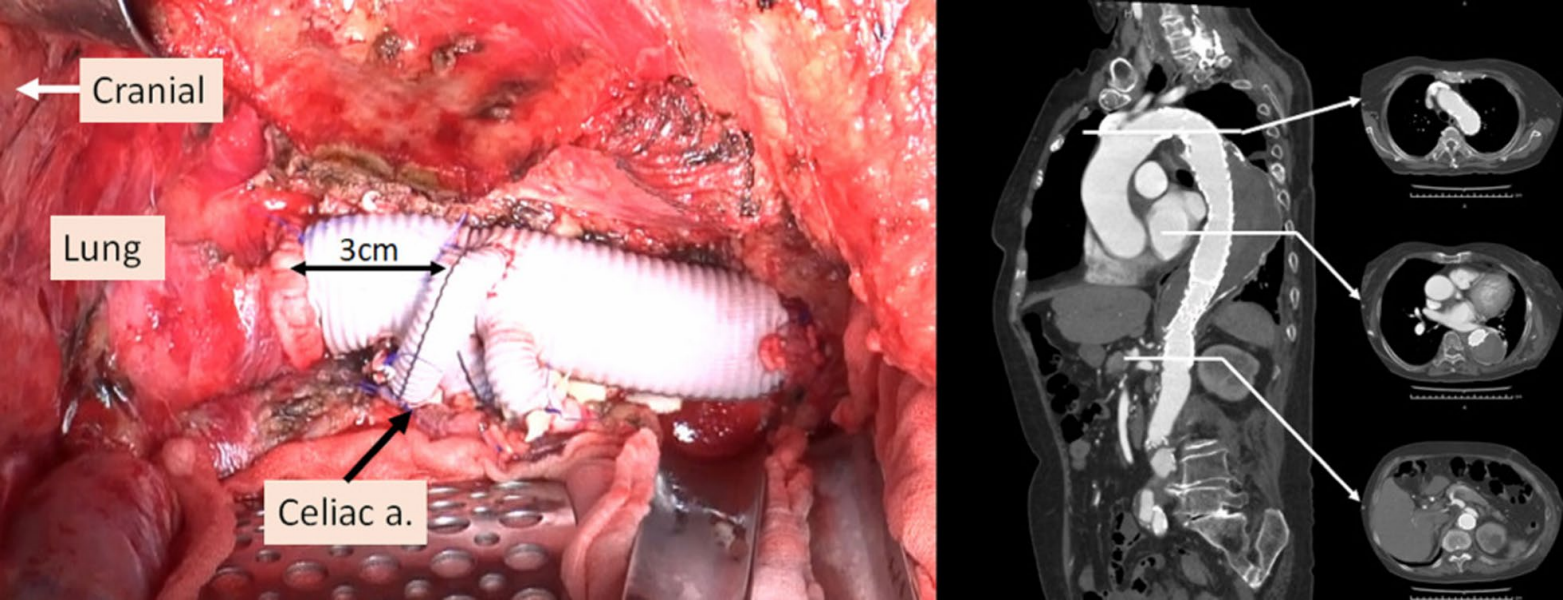
Intra-operative view through thoracoabdominal incision and postoperative enhanced computed tomography imaging. Left: The distance from the proximal anastomosis of the aorta to the branch for the celiac artery was adjusted to 3 cm to secure the distal landing zone for subsequent endovascular repair. Right: A CT scan, performed 1 month after the endovascular repair of the descending thoracic aorta, showed a faintly contrasted false lumen (type II endoleak)

Under deep hypothermic circulatory support, as in case 1, the aorta from immediately above the celiac artery to the bifurcation of the inferior mesenteric artery was replaced using a 24-mm four-branched vascular prosthesis (Gelweave Coselli Thoracoabdominal Graft, 24 mm, Vascutek Terumo Inc., Scotland, UK). In addition, the celiac, superior mesenteric, and right and left renal arteries were reconstructed. In this case, open proximal and distal anastomosis were performed using the double-barrel approach. The distance from the proximal aortic anastomosis to the branch for the celiac artery of the vascular graft was adjusted to 3 cm to secure a distal landing zone for the subsequent TEVAR (Fig. 5). The cardiopulmonary bypass, hypothermic Vf, retrograde cerebral perfusion, and operation times were 265, 196, 42, and 668 min, respectively.

TEVAR was performed 28 days after the procedure via the left common femoral artery. A 31 × 150-mm Conformable TAG (W. L. Gore & Associates, Inc., Flagstaff, AZ, USA) was deployed in the vascular prosthesis immediately proximal to the branch of the celiac artery. Next, a 34 × 200-mm Conformable TAG was deployed in the distal aortic arch immediately distal to the left subclavian artery to the first stent graft with sufficient overlap.

Her postoperative clinical course was uneventful, and she was discharged from hospital 9 days after TEVAR. One month after surgery, a CT scan showed type II endoleak; nevertheless, the patient was in good general condition (Fig. 5).

## Discussion and conclusion

Previous studies have established the usefulness of TEVAR in the management of acute type B aortic dissection [4, 5]. However, the treatment of cTBAD with TEVAR remains controversial for several reasons. Firstly, the intimal flap becomes thicker and stiffer, thus complicating the aortic remodeling. Secondly, blood flow into the false lumen from the remaining distal reentry exerts pressure on the false lumen, regardless of the presence of thrombosis. Finally, blood flow to the visceral artery from the false lumen may be reduced. Several methods, such as the candy-plug technique and Knickerbocker technique, have been developed to prevent blood reflux into the false lumen from the distal reentry [6, 7]. However, those methods may not consistently be successful in patients with cTBAD. Therefore, open repair is the most reliable and promising curative treatment for cTBAD.

As in the two cases described in the report, the presence of intra-pleural adhesions that are difficult to dissect may complicate the replacement of the aorta. Forcible dissection of these adhesions may result in lung injury, and such injury may lead to fatal postoperative complications (e.g., pulmonary hemorrhage, intractable pneumothorax, or pneumonia) [8]. In such situations, the surgeon may have to terminate the surgery and, consequently, rely on TEVAR. This is a regrettable situation for both the patient and surgeon. In such cases, replacement of the distal aorta, which is typically easy to access despite the presence of intra-pleural adhesions, with a vascular graft will serve as a reliable distal landing zone for subsequent TEVAR. This approach overcomes the limitations of TEVAR in the treatment of cTBAD.

Even in cases with severe adhesion on the diaphragmatic side of the lung, a thoracoabdominal incision with dissection of the costal arch may assist in securing the field of view and facilitate an adhesiotomy around the lung. In addition, access to the abdominal aorta through a thoracoabdominal incision is usually possible, regardless of the presence of pulmonary adhesions.

One of the disadvantages of this method is the risk of spinal cord injury (SCI). Cerebrospinal fluid drainage (CSFD) and mean arterial pressure augmentation are relatively effective methods for the prevention of SCI in thoracoabdominal aortic aneurysm (TAAA) repair. However, although CSFD is recommended as an effective method for the prevention of SCI in open TAAA repair, there is currently no strong evidence to support its effectiveness in TEVAR [9]. Because of the non-negligible rates of reported complications associated with CSFD, such as neuraxial hematoma, intracranial bleeding and meningitis, we do not routinely use CSFD.

Nakanishi et al. reported two patients with Marfan syndrome who had good outcomes [10]. Yamane et al. also reported using graft-to-graft bridging TEVAR for the dissection of a descending thoracic aortic aneurysm after aortic arch and thoracoabdominal placement in a patient with Marfan syndrome [11]. Both reports indicate that maintaining high blood pressure and hemoglobin levels postoperatively may reduce the risk of SCI. Therefore, in our two patients, we managed to maintain a mean postoperative blood pressure of ≥ 80 mmHg to prevent SCI. Fortunately, SCI was not observed in either case. However, the application of CSFD should be considered for patients with compromised spinal cord circulation, such as obstruction of the subclavian or vertebral arteries, or the internal iliac artery.

In the treatment of thoracoabdominal aortic aneurysm, use of a staged approach has been reported to be effective in preventing SCI [12]. However, there is no established evidence with regard to the adequate interval between open surgery and TEVAR. A longer interval carries the risk of rupture while waiting for TEVAR, while a shorter interval may lead to postoperative SCI due to insufficient development of a collateral network to the spinal cord. In a report of four cases of TAAA performed by Yoshida et al. using the same procedure as in the present report, three patients who underwent TEVAR between 19 and 48 days after open surgery had good outcomes without permanent SCI. However, one patient who was followed up after open surgery experienced a rupture 6 months later [13]. In another report on TAAA repair by Gombert et al., the interval between the two steps was 14.2 ± 15.3 days in six hybrid cases, and there was no occurrence of SCI [12]. In our department, TEVAR is performed approximately 2 weeks to 1 month after open surgery, because we think that this period allows sufficient time for the development of a collateral network to the spinal cord without increasing the risk of aneurysm rupture.

Unlike Case 2, the dissected thoracoabdominal aorta with visceral branches remained untouched in Case 1. This may cause subsequent aneurysmal enlargement of the aorta, which can require additional surgery. Therefore, Crawford type IV replacement appears to be the most suitable method to create a distal landing zone and avoid additional surgery on the distal aorta. However, although Case 1 was a relatively young patient, Crawford type IV replacement was not selected because of preoperative left hemiparesis, the presence of critical intercostal artery in Th12, and the relatively small maximum diameter of this area (i.e., 38 mm). In case of future open surgery for distal aortic lesion, the vascular prosthesis would be covered by the native aortic wall or the pleura to prevent direct adhesion between the lung and the vascular prosthesis as in Case 1.

Another disadvantage of this method is the risk of type II endoleak. Thus, long-term follow-up is necessary because type II endoleak may lead to aneurysm formation in the false lumen.

Another method of TEVAR after distal fenestration has also been reported [14, 15]. However, in all these methods, the dissected native aortic wall becomes the distal landing zone. Hence, there is concern regarding the occurrence of subsequent stent graft-induced new entry, or aneurysmal dilatation around the distal landing zone leading to type Ib endoleak. The present method, in which the vascular graft is used as a landing zone, may eliminate these concerns.

Since open surgical repair of thoracoabdominal aorta continues to be an exceptionally high-risk surgery, TEVAR would be an option for Case 2, a relatively elderly woman over 80 years of age. However, this patient was still active and had normal cognitive function, despite her advanced age. Moreover, in accordance with the statistics from the Ministry of Health, Labour and Welfare in Japan, the average life expectancy for an 80-year-old woman is over 12 years. Based on this information, as well as the patient’s request, we decided to perform open surgical repair on this patient.

This method may be a viable option for the management of severe adhesions accidentally detected in the pleural cavity during open repair for cTBAD.

## Data Availability

The datasets used in this report are available from the corresponding author upon reasonable request.

## Abbreviations

CSFD: Cerebrospinal fluid drainage
CT: Computed tomography
cTBAD: Chronic type B aortic dissection
SCI: Spinal cord injury
TAAA: Thoracoabdominal aortic aneurysm
TEVAR: Thoracic endovascular aortic repair
